# The Interplay of Disability, Depression, Social Support, and Quality of Life in Middle-Aged and Young Couples Affected by Stroke: A Dyadic Path Analysis Using the Actor–Partner Interdependence Mediation Model

**DOI:** 10.3390/nursrep15100372

**Published:** 2025-10-20

**Authors:** Ya-Ting Liu, Dan-Dan Xiang, Song Ge, Shan-Shan Wang, Jun-Fang Xie, Zhi-Wei Liu, Si-Xun Zhang, Zhen-Xiang Zhang, Su-Yan Chen, Xin Li, Yong-Xia Mei

**Affiliations:** 1 Department of Nursing, School of Nursing and Health, Zhengzhou University, 101 Science Avenue, Zhengzhou 450001, China; lyt17699332978@163.com (Y.-T.L.); liuzhiwei@gs.zzu.edu.cn (Z.-W.L.); zsx88@gs.zzu.edu.cn (S.-X.Z.); zhangzx6666@126.com (Z.-X.Z.); 2 Department of Nursing, Zhengzhou Shuqing Medical College, Zhengzhou 450064, China; 3 Natural Science Department, University of Houston-Downtown, Houston, TX 77002, USA; ges@uhd.edu; 4 Department of Nursing, School of Nursing, The Hong Kong Polytechnic University, Hong Kong, China; shan-shan.wang@polyu.edu.hk; 5 Department of Gynecology, The Third Affiliated Hospital of Zhengzhou University, Zhengzhou 450052, China; xiejunfang2011@163.com; 6 Department of Neurology, The Second Affiliated Hospital of Zhengzhou University, Zhengzhou 450014, China; lizn999@126.com

**Keywords:** stroke, caregiver, mental health, quality of life, nursing

## Abstract

**Objective:** The purpose of this study was to explore the impact of disability on dyadic quality of life (QoL) among stroke survivors and to examine the mediating role of social support in this process. **Methods:** Outcome measures were collected at four time points: baseline, 1 month, 3 months, and 6 months post-discharge. The Actor–Partner Interdependence Mediation Model was used to analyze the dyadic data. **Results:** A significant association was observed between a higher degree of disability and more severe depressive symptoms in stroke survivors (*β* = 0.626) and their spouses (*β* = 0.426). Survivors’ disability had a negative impact on their own physical health (*β* = −3.731) and indirectly affected the physical health of the spouse caregiver through the spouse caregiver’s depression (*β* = −0.198). In addition, disability affects the survivor’s own mental health through depression and social support (*β* = −0.231) and indirectly through the spouse caregiver’s depression and their own social support (*β* = −0.156). **Conclusions:** Survivor disability has a major impact on depression and QoL in couples with stroke. It is recommended that healthcare providers should identify disability early in stroke survivors and then target interventions to improve the QoL of couples affected by stroke who are at high risk of negative emotions.

## 1. Introduction

Stroke is the second leading cause of mortality and the leading cause of adult disability worldwide [1]. It is concerning that the overall incidence of stroke has shifted toward younger age groups [2]. The American Heart and Stroke Association has reported that approximately 10% of stroke cases occur in individuals aged 18 to 50 [3]. Data from the Global Burden of Disease (GBD) study further indicates that the incidence of stroke among individuals aged 15–39 has risen steadily from 1990 to 2019 [4]. In China, the China Stroke Surveillance Report 2021 indicates that the average age of stroke onset is gradually decreasing, with individuals aged 40–64 now accounting for over 66.6% of first-time stroke cases [5]. Compared to elderly stroke survivors, the harm caused by this ‘younger onset’ trend is particularly significant. This demographic is often in the ascendant phase of their careers, bearing multiple roles and responsibilities within families and society. Once a stroke occurs, the survivor’s life is significantly disrupted, and a series of cascading effects may ensue [2]. Studies have shown that post-stroke survivors’ independence in daily living and levels of depression and anxiety is strongly associated with their own quality of life (QoL) [4]. At the same time, the QoL of post-stroke survivors was significantly correlated with caregiver anxiety, depression and caregiving burden, and when the QoL of survivors declined, the physical health of caregivers was also affected [5].

According to research, 75% to 80% of stroke survivors experience some degree of disability after their stroke [6]. The sudden decline in physiological functioning severely influences the mental health of the patient, and approximately one-third of stroke survivors may experience depressive symptoms [7]. In China, a cross-sectional studies have further shown that disability is a significant predictor of post-stroke depression [8]. A follow-up study of stroke survivors in young and middle-aged adults revealed that 44.7% of stroke survivors exhibited a poor functional prognosis, which significantly impacts the survivor’s QoL [9]. However, existing studies have primarily concentrated on the identification of the most prevalent physical disabilities and their associated distress among stroke survivors. There is little we know about exploring the relationship between stroke survivors’ impairments and the health outcomes of spousal caregivers.

As a severely disabling condition, stroke faces a protracted period of rehabilitation. In China, due to strong family values, spouses frequently serve as primary caregivers post-discharge [10]. This sudden caregiving role shift can increase the risk of depressive symptoms, with a reported prevalence of 53.9% among spousal caregivers [11]. In comparison to older survivors, younger and middle-aged survivors are more likely to experience depression due to the additional economic, familial, and social pressures they face [12]. The prevalences of depression, anxiety, and stress in the post-stroke population have been reported as 74.5%, 52.9%, and 68%, respectively [13]. A systematic review also noted [14] that the overall prevalence of post-stroke depression was 27%. Of those who developed depression within 3 months of stroke, 53% suffered from persistent depression, and the cumulative prevalence remained as high as 38% at 1 year post-stroke. Such negative emotions may also result in a vicious cycle of diminished QoL and heightened adverse disease events, which in turn impede the recovery process.

Social support refers to the emotional, informational, and practical assistance individuals perceive from others in daily life [15]. Research indicates that social support significantly impacts stroke patients’ QoL, particularly emotional support, which effectively alleviates depressive symptoms and enhances functional recovery [16]. According to the Stress Buffering Hypothesis, social support indirectly promotes stroke survivors’ quality of life by reducing stress responses, alleviating depressive symptoms, and enhancing rehabilitation motivation [17]. The family, as the most fundamental social unit for patients, represents the most direct and common form of social support, providing both practical care and emotional fulfillment. Research indicates that high-quality emotional support from spouses, relatives, and friends can reduce survivors’ depression levels and improve activities of daily living [18]. Medical-social support promotes patient health through regular follow-up, rehabilitation guidance, and psychological interventions. However, studies have found that informal caregivers have the highest levels of depression and the most lack of medical social support during the acute phase of stroke [19]. In addition, the shortage of rehabilitation resources in remote areas of China makes it difficult for stroke survivors to access structured rehabilitation services [20]. Compared to older survivors, young and middle-aged stroke survivors face greater role conflicts from work and family demands [21], while caregivers risk marginalization and exhaustion due to prolonged caregiving and disrupted social networks [22]. Therefore, optimizing the social support system for young and middle-aged stroke survivor couples to enhance their long-term QoL is of great practical importance.

The trajectory model of chronic disease posits that the development of chronic disease is a protracted process, with disparate trajectories emerging from varying survivor physical conditions and distinct disease stages [23]. Kirkevold et al. proposed four phases of recovery after stroke: the onset period (1–7 days after stroke), the early recovery period (1–8 weeks after stroke), the period of sustained recovery (8 weeks to 6 months after stroke), and the period of semi-stability (6–12 months after stroke) [24]. Considering that the most substantial recovery takes place within the initial six months, it is of paramount importance to investigate the interactions among disability, depression, social support, and QoL during this pivotal period. Consequently, this study carried out longitudinal follow-up surveys at four time points: within 1–2 days before discharge (T0), 1 month after discharge (T1), 3 months after discharge (T2), and 6 months after discharge (T3).

This study is based on the Dyadic Illness Management Theory (DIMT) and the Actor–Partner Interdependence Mediation Model (APIMeM) for theoretical framework and hypothesis derivation. DIMT emphasizes that patient and caregiver health states are intertwined and affect each other, requiring simultaneous attention and intervention [25]. Cohort studies including Kruithof found that social support significantly declines over time among stroke patients, and this decline is associated with prior depressive symptoms [26]. Evidence suggests that depression in stroke survivors increases caregivers’ risk of depression and erodes their social support networks over time, while caregiver fatigue further limits support provision [27]. However, most prior studies have focused separately on either stroke survivors or caregivers, leaving insufficient evidence on how survivors’ functional impairments influence caregivers’ health outcomes through psychological and social mechanisms. Thus, we propose the pathway hypothesis: T0 disability → T1 depression → T2 social support. Furthermore, depression induces cumulative physiological and psychological effects—such as inflammatory responses, neuroendocrine dysregulation, and diminished self-efficacy—ultimately compromising overall health [28]. This supports the hypothesis: T0 disability → T1 depression → T2 social support → T3 physical and mental health (with depression mediating the relationship between disability and quality of life, and the moderating role of social support). However, while the DIMT emphasizes the relational and interactive nature of the patient-caregiver dyad, it falls short at the statistical modeling level in precisely distinguishing individual effects from partner effects. To address this limitation, the APIMeM provides a crucial tool for dyadic research. It distinguishes between the actor’s effect on their own outcome (actor effect) and on their partner’s outcome (partner effect), while simultaneously testing direct and indirect effects between individual and partner variables to uncover mediating mechanisms within the dyad [29]. Therefore, this study employs DIMT as its theoretical foundation and leverages the methodological strengths of APIMeM to validate path effects of interactions within dyadic relationships.

The objective of this study was (1) to examine the dynamics of disability over time in stroke survivors and to explore how this change affects couple dyadic QoL (physical and mental health) in young and middle-aged stroke survivors (2) To explore the impact of disability, depression, and social support on the dyadic physical and mental health of young and middle-aged stroke couples, and the mediating role of social support.

## 2. Methods

### 2.1. Design

The research employed a longitudinal study design grounded in the actor–partner interdependence mediation model (APIMeM). The reporting of this study followed the Strengthening the Reporting of Observational studies in Epidemiology (STROBE) checklist (Table S1).

### 2.2. Participants

Young and middle-aged stroke couples were recruited using convenience sampling methods. The inclusion criteria for stroke survivors were: (1) First-time stroke confirmed by CT or MRI; (2) Age: females 20–59, males 22–59 (according to China’s legal age for marriage); (3) Stable vital and need for caregiver assistance; (4) Intact language and cognition, with informed consent. Inclusion criteria for caregivers were: (1) Spouse of the stroke survivor; (2) Provided ≥4 h of daily care on average; (3) Age: females ≥ 20, males ≥ 22; (4) Intact language and cognition, with informed consent. Exclusion criteria for both: (1) Severe chronic illnesses (e.g., cancer, heart/kidney/respiratory failure); (2) Participation in other psychological intervention.

### 2.3. Procedure of Data Collection

Stroke couples were recruited from neurology departments of three Grade IIIA hospitals in Henan Province, China, from July 2022 to October 2023. Informed consent was obtained after explaining the study purpose and procedures. Baseline data (T0) were collected via questionnaires administered individually within hospital rooms to prevent interference. Follow-up data were gathered over the telephone at 3 months (T1), 6 months (T2), and 12 months (T3) post-discharge. A follow-up schedule was established based on the initial baseline collection time to ensure timely and comprehensive follow-up. A total of 168 stroke survivor couples ultimately participated in this study. After each follow-up, participants received a health assessment report as appreciation.

### 2.4. Instruments

A demographic questionnaire was designed by researchers to collect information on stroke survivors, including age, gender, education level, payment method of medical expenses, average monthly family income, occupation, disease type, symptoms, complications, whether the family was the main economic support and recurrent times during follow-ups. For caregivers, data included age, gender, education, occupation, daily care time, and presence of chronic diseases.

The modified Rankin Scale (mRS) is a widely employed assessment tool used to gauge neurological functional recovery following a stroke [30]. This scale evaluates the degree of disability and functional independence after a stroke, ranging from 0 (no symptoms) to 5 (severe disability). The Cronbach’s α coefficient for this scale in this study was 0.756.

Depression in stroke survivor couples was assessed using the Chinese version of the 9-item Patient Health Questionnaire (PHQ-9), with each item rated on a 4-point Likert scale (total score 0–27). Higher scores indicate more severe depression [31]. In this study, Cronbach’s α was 0.869 for survivors and 0.706 for caregivers.

Perceived social support was measured using the Chinese version of the Perceived Social Support Scale [32], which includes 12 items across two dimensions (in-family and out-of-family support) rated on a 7-point scale. Scores range from 0 to 84, with higher scores reflecting greater support. In this study, Cronbach’s α for stroke survivors and their spouse caregivers were 0.914 and 0.879, respectively.

The Chinese version of the 12-item Short-Form Health Survey Scale was utilized to assess the QoL among couples of stroke survivors, evaluating their dyadic health levels [33]. The first four domains are physical component scores (PCS) and the remaining four domains are mental component scores (MCS). The raw scores of the entries were first converted and then the raw scores were converted to standardized values according to the weight factor table. In the current study, the Cronbach’s α values were found to be 0.899 for stroke survivors and 0.727 for their spouse caregivers.

### 2.5. Data Analysis

According to Ledermann et al., for the APIMeM, a sample size of approximately 120 dyads is required to ensure sufficient statistical power for testing mediation effects [29]. Considering stroke survivors and their spouses as distinguishable dyadic relationships, the medium effect was set at 0.25, statistical power at 0.80, correlation of the actor and partner variables at 0.3, correlation of the errors at 0.3, and two-tailed significance level α = 0.05, the required sample size was 121 dyads. Allowing for a 20% attrition rate, 168 stroke survivor couples were finally recruited.

IBM SPSS 29.0 was used for general data description of stroke survivor couples. Descriptive statistics such as means with standard deviation for normally distributed continuous data, medians with interquartile range for non-normally distributed continuous data, and frequency with percentages for categorical data were employed. *χ*^2^ test was applied for categorical data, and independent sample *t*-test for continuous data to compare general data between spouses of stroke survivors who completed follow-up and those who were lost to follow-up. Generalized Estimating Equation was used for count data, and Repeated Measures Analysis of Variance for measurement data to compare differences between variables at each time point.

Amos 24.0 was employed for constructing the APIMeM. The model was parameterized using Maximum Likelihood Estimation and the model fit was assessed by a number of fit indices, including the Chi-square (*χ*^2^), Degrees of Freedom (*df*), Root Mean Square Error of Approximation (RMSEA), Goodness of Fit Index (GFI), Incremental Fit Index (IFI), Comparative Fit Index (CFI), and Tucker–Lewis Index (TLI) were used to evaluate model fit. Criteria for a good fit included *χ*^2^/*df* < 5, RMSEA ≤ 0.08, GFI ≥ 0.90, IFI ≥ 0.90, CFI ≥ 0.90, TLI ≥ 0.90 [34]. The Deviation-Corrected Percentile Bootstrap method (sampling 5000 samples) was employed to test the mediating effect, with a Bootstrap 95% Confidence Interval (Bootstrap 95% *CI*) without 0 to judge the existence of a mediating effect.

### 2.6. Ethical Considerations

This study has obtained approval from the Life Sciences Ethics Review Committee of Zhengzhou University, with the ethics number: ZZUIRB2020-53. Prior to the research investigation, both partners in the couples of stroke survivors were required to provide written informed consent after being fully informed about the study.

## 3. Results

### 3.1. Demographic Characteristics of the Dyadic

**Figure 1:**
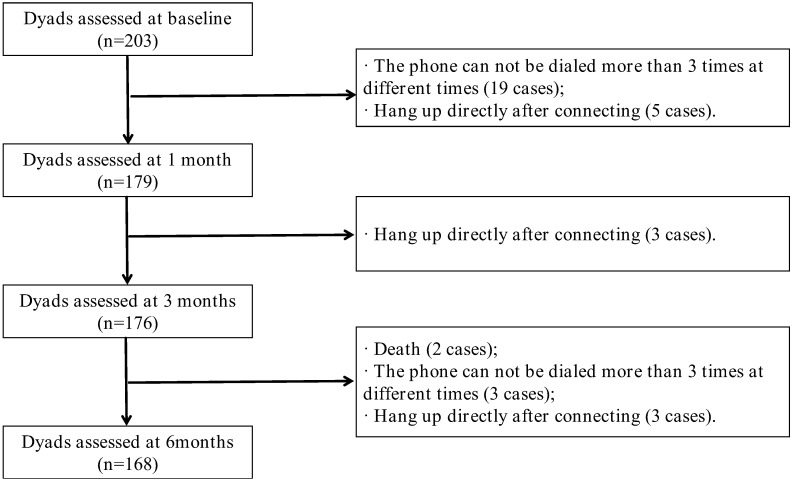
Flow chart of the study.

The study comprised 168 pairs within its study population. The flow chart of study inclusion and follow-up was shown in Figure 1.

Most survivors were male with cerebral infarction, and 76.2% had comorbidities; 3.94% experienced relapse during follow-up. Spouse caregivers had an average age of 50.77 (7.38) years, were mostly female, and 24.4% had chronic illnesses. Detailed sociodemographic data are presented in Table 1. Regarding the follow-up, a total of 35 couples were lost to follow-up. Additional specifics can be found in Table 1.

### 3.2. Variable Scores and Trends over Time Among Stroke Survivor Couples

The incidence of poor prognosis among survivors decreased from 31.0% at T0 to 10.7% at T3, indicating an overall declining trend (Wald *χ*^2^ = 42.36, *p* < 0.001). Pairwise comparisons revealed significant differences between T0–T1 (*p* = 0.002) and T1–T2 (*p* = 0.013), but not between T2–T3 (*p* = 0.264).

Depression scores in survivors decreased from 5.55 (4.25) at T0 to 1.96 (3.12) at T3 (F = 36.871, *p* < 0.001). Caregivers’ depression scores also declined from 3.21 (2.41) to 0.94 (1.49) (F = 49.967, *p* < 0.001).

Social support scores showed no significant overall change in either survivors (from 56.23 [11.25] to 54.78 [9.68], F = 1.744, *p* = 0.106) or caregivers (from 57.52 [8.33] to 56.63 [7.78], F = 2.362, *p* = 0.073).

Stroke survivors showed improved physical health from T0 34.47 (8.41) to T3 46.44 (8.05) (F = 117.838, *p* < 0.001), and mental health increased from 45.26 (7.35) to 52.54 (8.38) (F = 79.175, *p* < 0.001). Spouse caregivers’ physical health slightly declined from 53.52 (3.77) to 53.11 (3.28) with a significant overall change (F = 3.677, *p* = 0.013), while their mental health improved from 49.18 (5.77) to 54.47 (5.34) (F = 54.986, *p* < 0.001).

### 3.3. The Mediating Effect of Dyadic of Physical Health and Disability-Depression-Social Support in Couples of Stroke Survivors

Based on the theoretical framework and clinical significance of this study, the dyadic physical health incorporating disability degree at T0, depression at T1, and social support at T2 and T3 was utilized to construct an APIMeM for stroke survivor couples. The final model exhibited a good fit with *χ*^2^ = 4.968, *df* = 5, *χ*^2^/*df* = 0.994, GFI = 0.991, IFI = 1.000, TLI = 1.000, CFI = 1.000, and RMSEA < 0.001.

**Figure 2:**
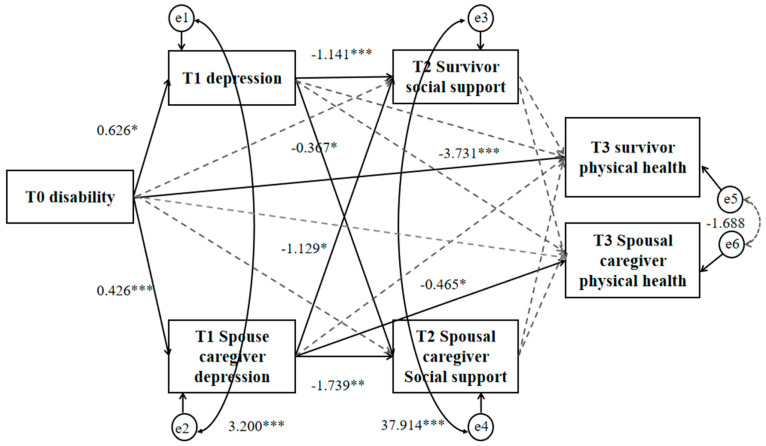
The actor and partner mediating effect of dyadic physical health and disability–depression–social support. Note: * *p* < 0.05, ** *p* < 0.01, *** *p* < 0.001, 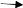 path significant, 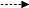 path not significant.

The degree of disability among survivors emerged as a direct positive predictor of depression (*β* = 0.626, *p* < 0.05) and the depression experienced by their spouse caregivers (*β* = 0.426, *p* < 0.05). Additionally, we found that the degree of disability in survivors directly negatively impacted their own physical health (β = −3.731, *p* < 0.05). The survivor’s level of depression not only significantly negatively predicted their own social support (β = −1.141, *p* < 0.05), but also negatively affected the social support of the spouse caregiver (β = −0.367, *p* < 0.05). Furthermore, the degree of disability among survivors indirectly influenced the physical health of spouse caregivers through the depression experienced by the caregivers (*β* = −0.198, *p* < 0.05), illustrated in Figure 2 and Table 2. Table 2 presents only statistically significant (*p* < 0.05) effect results. Complete effect results are detailed in Table S2.

### 3.4. The Mediating Effect of Dyadic Mental Health and Disability–Depression–Social Support in Couples of Stroke Survivors

In alignment with the theoretical framework and clinical significance of this study, the interplay between disability at T0, depression at T1, and social support at T2 and T3 pertaining to mental health were employed to establish a dyadic model for mental health concerning disability, depression, and social support for stroke survivor couples. The final model fits good with *χ*^2^ = 5.629, *df* = 6, *χ*^2^/*df* = 0.938, GFI = 0.990, IFI = 1.001, TLI = 1.003, CFI = 1.000, and RMSEA < 0.001.

**Figure 3:**
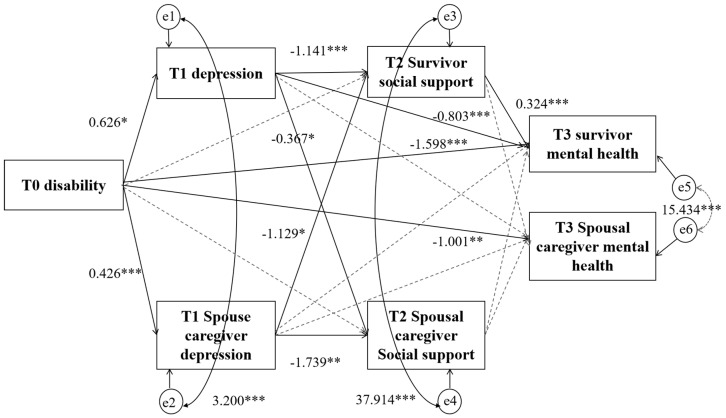
The actor and partner mediating effect of dyadic mental health and disability–depression–social support. Note: * *p* < 0.05, ** *p* < 0.01, *** *p* < 0.001, 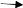 path significant, 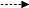 path not significant.

The degree of disability among survivors emerged as a direct positive predictor of depression (*β* = 0.626, *p* < 0.05) experienced by both the survivors and their spouse caregivers (*β* = 0.426, *p* < 0.05). Additionally, the degree of disability in survivors directly and negatively impacted both their own mental health (*β* = −1.598, *p* < 0.05) and that of their spouse caregivers (*β* = −1.001, *p* < 0.05). Furthermore, the disability degree in survivors exhibited indirect effects on their mental health through their own depression and social support (*β* = −0.231, *p* < 0.05), and similarly through the depression and social support experienced by their spouse caregivers (*β* = −0.156, *p* < 0.05). Refer to Figure 3 and Table 3. Table 3 presents only statistically significant (*p* < 0.05) effect results. Complete effect results are detailed in Table S3.

## 4. Discussions

The aim of this study was to examine the longitudinal trajectory of disability and its impact on stroke couples’ QoL in young and middle-aged stroke survivors, and to further explore the mediating role of social support in this process. The findings of this study indicate that the disability levels of stroke survivors generally decline over time. Concurrently, improvements in disability levels are negatively correlated with the physical and mental health of both patients and their spouses, that is, higher disability levels correlate with poorer physical and psychological health among both patients and their spouses. Notably, the disability level of stroke survivors not only directly impacts their own physical health but also adversely affects their long-term physical health by increasing the depression levels of their spouse caregivers, revealing a potential chain reaction between disability and caregiver health. Furthermore, the depression experienced by spouse caregivers not only negatively affects their own psychological well-being but also exerts a cross-actor effect on the psychological health of patients by diminishing the patients’ perceived social support. These findings further reveal the interdependence of couple dyadic health QoL in stroke survivors and emphasize the integration of psychological support and social resources into stroke rehabilitation programs to enhance dyadic health quality.

### 4.1. Effects of Disability on Dyadic Physical Health

The present study found that the level of disability of stroke survivors has a direct impact on the survivors’ independence in daily life, which in turn has a significant impact on their physical health. This is in line with the findings of Chang et al. who found that the level of disability of stroke survivors was negatively correlated with their own QoL within three months of stroke [35]. This is further validated by a study by Chuluunbaatar et al. which demonstrated a strong correlation between stroke survivors’ decreased ability to perform daily living tasks and their decreased QoL trajectory [36]. Stroke survivors’ level of disability (T0) not only directly affects their own physical health, but also negatively impacts caregivers’ long-term (T3) physical health through spousal caregiver depression levels (T1) and ultimately, caregivers’ long-term (T3) physical health. It is suggested that the patient’s functional impairment has a delayed impact on the caregiver’s physical health, which may be closely related to the long-term accumulation of the caregiver’s psychological stress and caregiving burden. This is similar to the findings of Pont et al., who found that 11.1% of caregivers of stroke survivors noted that their caregiving burden was more likely to change and be heavy at 6 or 12 months [37]. As the length of caregiving progresses, caregivers may experience a gradual buildup of psychological stress, becoming more susceptible to anxiety and depression, and negatively impacting their own health through a range of physiological mechanisms [38]. The reason may be that, compared to elderly patients, young and middle-aged patients are typically at the peak of their careers, bear heavier family responsibilities, and have greater needs for rehabilitation and social participation [37]. During the transition period from hospital discharge to home-based rehabilitation, caregivers often need to rapidly adapt to their new caregiving role. They not only shoulder significantly increased caregiving tasks and emotional support but may also face financial pressures and conflicts with their own work commitments [30]. This disparity suggests that clinical intervention strategies should be tailored to patients’ age and life role characteristics. Furthermore, attention should extend beyond functional recovery to include psychological support and health management for caregivers, thereby optimizing dyadic health outcomes.

### 4.2. Effects of Depression on Dyadic Physical Health

The study revealed a decline in depressive symptoms among both stroke survivors and their spousal caregivers over time. Notably, at baseline, stroke survivors exhibited higher depressive symptoms compared to their spousal caregivers, a trend that remained consistent even after 6 months. Consistent with Pan et al. findings, the longitudinal alterations in the physical health of stroke survivors were solely associated with a negative correlation with the survivors’ depression [39]. This potential associations might be attributed to the depressive symptoms hindering the survivors’ enthusiasm to engage in treatment and rehabilitation, subsequently impeding their overall recovery and daily functioning, leading to a decline in physical health [40]. Moreover, survivors’ depression often increases the burden on caregivers, thereby exacerbating the caregivers’ physical health status [41]. This study reaffirms the detrimental impact of depression on the dyadic physiological health of stroke survivors. Consequently, it suggests that systematic depression screening should be integrated into routine clinical assessments for stroke rehabilitation. For instance, conducting dynamic evaluations of depression levels in both patients and their spouses one month post-discharge facilitates early identification of high-risk couple dyadic health, thereby enabling coordinated improvements in dyadic health outcomes.

### 4.3. The Mediating Role of Social Support in the Relationship Between Disability and Dyadic Health

The results of the present study suggest that the level of disability may also negatively affect the mental health of stroke survivors (T3) by influencing the level of depression in spousal caregivers (T1), indirectly decreasing the patient’s level of social support (T2), and ultimately, the mental health of stroke survivors (T3). Previous studies have noted that the prevalence of depression in stroke caregivers is as high as 30–40% and is closely related to the patient’s level of functional impairment [27]. When caregivers are depressed, they tend to reduce emotional communication and interaction with the patient, weakening the quality of intimacy and even triggering family tensions, resulting in a decline in the patient’s perceived family support [38]. Social support is an important protective factor for the functional recovery and psychological adaptation of stroke patients, whereas caregivers in a depressed state may display characteristics such as indifference and lack of patience, reduce companionship and encouragement to patients, which in turn inhibits their social participation, limits access to support resources, and ultimately exacerbates the psychological distress of patients [42].

In addition, the level of depression in caregivers not only affects the mental health of survivors, but may further impact the long-term mental health of caregivers by reducing the level of social support for survivors. Previous studies have shown that adequate social support not only helps patients’ functional recovery and psychological adjustment, but also relieves caregivers’ psychological stress [27]. However, the present study found that when survivors’ perceived social support gradually declined, their social support network also further atrophied, which not only aggravated the survivors’ psychological burden but also led to deterioration of the caregivers’ mental health. This finding is consistent with the findings of Bartoli et al. that caregivers’ psychological status is relatively stable in the early stages of stroke rehabilitation, but their levels of depression and anxiety may further increase as rehabilitation progresses slowly [42]. Therefore, dyadic health outcomes can be optimized during stroke rehabilitation by improving family-based communication models, facilitating patient participation in community-based rehabilitation groups, and optimizing telepsychological counseling.

### 4.4. Limitations

The study has several limitations: (1) Convenience sampling was employed, with research subjects limited to Henan Province, which may restrict sample representativeness. Furthermore, cultural expectations surrounding family caregiving roles in China differ from those in Western individualistic societies. In China, greater emphasis is placed on family responsibilities, filial piety, and collectivist values. These cultural specificities may limit the cross-cultural generalizability of the findings. (2) The study used self-assessment scales, which may be affected by participants’ subjective judgment or memory bias; (3) Although the sample size of this study meets the statistical requirements for testing the primary model, the overall scale remains relatively limited, which may affect the stability and generalizability of the results to some extent. Future research could be conducted in a multicenter setting and further expand the sample size to enhance the robustness and generalization value of the findings.

## 5. Conclusions

A dyadic effect exists for stroke survivors and their spouse caregivers. The survivor’s dysfunction not only directly impairs his or her own mental health but also indirectly diminishes the patient’s perception of social support by increasing the level of depression in the spousal caregiver, which ultimately has a negative impact on the couple’s physical health. Research further suggests that social support plays a mediating role in this process, and that its level is moderated not only by the degree of disability of the survivor, but also by the psychological state of the caregiver.

## Figures and Tables

**Table 1:** Comparison of characteristics of stroke survivors-caregivers in follow-up group and drop-out group [n (%)].

Characteristic		Follow-Up (N = 168)	Drop-Out (N = 35)	Statistic	*p*-Value
Stroke Survivors					
Age ($\bar{x}$ ± s)		51.32 ± 6.90	50.94 ± 7.11	0.289	0.773 ^c^
Gender				0.038	>0.999 ^a^
Male		127 (75.6)	27 (77.1)		
Female		41 (24.4)	8 (22.9)		
Education				0.180	0.945 ^a^
Primary school		43 (25.6)	10 (28.6)		
Middle school		68 (40.5)	13 (37.1)		
High school		57 (33.9)	12 (34.3)		
Monthly income (Yuan)				0.447	0.843 ^a^
<3000		65 (38.7)	12 (34.3)		
3000~5000		59 (35.1)	12 (34.3)		
>5000		44 (26.2)	11 (31.4)		
Occupation before illness				-	0.309 ^b^
Farmer		46 (27.4)	7 (20.0)		
Worker		45 (26.8)	15 (42.9)		
Technical staff		31 (18.5)	8 (22.9)		
Merchant		23 (13.7)	2 (5.7)		
others		17 (10.5)	3 (8.6)		
Medical payment form				-	0.391 ^b^
Employee health insurance		42 (25.0)	12 (34.3)		
Resident medical insurance		115 (68.5)	20 (57.1)		
others		11 (6.5)	3 (8.6)		
Family the main economic pillar				0.035	>0.999 ^a^
Yes		132 (78.6)	27 (77.1)		
No		36 (21.4)	8 (22.9)		
Stroke type				-	0.814 ^b^
hemorrhagic		12 (7.1)	3 (8.6)		
ischemic		154 (91.7)	32 (91.4)		
Mixed type		2 (1.2)	0 (0.0)		
Number of complications				0.236	0.669 ^a^
None		40 (23.8)	7 (20.0)		
1 or more kinds		128 (76.2)	28 (80.0)		
Main symptom				0.571	0.538 ^a^
Limb dysfunction		119 (70.8)	27 (77.1)		
others		49 (29.2)	8 (22.9)		
Caregivers					
Age ($\bar{x}$ ± s)		50.77 ± 7.38	51.29 ± 6.99	−0.381	0.704 ^c^
Gender				0.038	>0.999 ^a^
Male		41 (24.4)	8 (22.9)		
Female		127 (75.6)	27 (77.1)		
Education				5.691	0.065 ^a^
Primary school		47 (28.0)	17 (48.6)		
Middle school		74 (44.0)	11 (31.4)		
High school		47 (28.0)	7 (20.0)		
Occupation				-	0.079 ^b^
Farmer		43 (25.6)	5 (14.3)		
Worker		41 (24.4)	14 (40.0)		
Technical staff		22 (13.1)	1 (2.9)		
Merchant		17 (10.1)	2 (5.7)		
others		45 (26.8)	13 (37.1)		
Have a chronic disease				1.692	0.267 ^a^
Yes		41 (24.4)	5 (14.3)		
No		127 (75.6)	30 (85.7)		

Note: *p* Value was a comparison between the follow-up group (N = 168) and the drop-out group (N = 35) (excluding selection bias); ^a^: *χ*^2^ test; ^b^: Fisher’s precision probability test; ^c^: Two independent samples *t* test.

**Table 2:** The dyadic PCS and disability–depression–social support chain mediation effect (N = 168).

Effect	β	SE	95%*CI*	*p*-Value
The actor effect of survivors				
Direct effect	−3.731	0.589	−5.014, −2.669	0.001
Total effect	−0.817	0.553	−5.219, −3.052	0.001
Partner effects of spouse caregivers				
Indirect effect				
T0 Disability 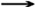 T1 Spouse caregiver depression 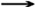 T3 Spouse caregiver PCS	−0.037	0.106	−0.468, −0.038	0.011
Total effect	−0.092	0.231	−0.946, −0.057	0.030

**Table 3:** The dyadic MCS and disability–depression–social support chain mediation effect (N = 168).

Effect	β	SE	95%*CI*	*p*-Value
The actor effect of survivors				
Direct effect	−1.598	0.530	−2.732, −0.641	0.001
Indirect effect				
T0 Disability 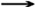 T1 Survivor depression 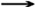 T3 Survivor MCS	−0.104	0.287	−1.279, −0.096	0.007
T0 Disability 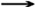 T1 Survivor depression 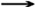 T2 Survivor social support 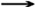 T3 Survivor MCS	−0.462	0.134	−0.643, −0.062	0.003
T0 Disability 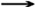 T1 Spouse caregiver depression 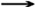 T2 Survivor social support 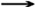 T3 Survivor MCS	−0.312	0.117	−0.515, −0.023	0.012
Total effect	−2.439	0.621	−3.458, −1.007	0.001
Partner effects of spouse caregivers				
Direct effect	−1.001	0.351	−1.727, −0.325	0.003
Indirect effect				
T0 Disability 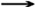 T1 Survivor depression 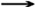 T2 Survivor social support 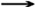 T3 Spouse caregiver MCS	−0.566	0.050	−0.207, 0.000	0.049
T0 Disability 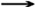 T1 Spouse caregiver depression 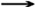 T2 Survivor social support 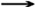 T3 Spouse caregiver MCS	−0.306	0.036	−0.163, −0.002	0.038
Total effect	−1.896	0.412	−2.261, −0.650	0.001

## Data Availability

The data supporting the findings of this study are available on request from the corresponding author. The data are not publicly available due to privacy or ethical restrictions.

## Supplementary Materials

The following supporting information can be downloaded at: https://www.mdpi.com/article/10.3390/nursrep15100372/s1, Table S1. STROBE Statement—Checklist of items that should be included in reports of cohort studies. Table S2. The Dyadic PCS and disability–depression–social support chain mediation effect (N = 168). Table S3. The Dyadic MCS and disability–depression–social support chain mediation effect (N = 168).

## Public Involvement Statement

No public involvement in any aspect of this research.

## Guidelines and Standards Statement

This study was reported in accordance with the Strengthening the Reporting of Observational Studies in Epidemiology (STROBE) Statement, which is applicable to longitudinal observational studies.
