# Supplementary material for: The Interplay of Disability, Depression, Social Support, and Quality of Life in Middle-Aged and Young Couples Affected by Stroke: A Dyadic Path Analysis Using the Actor–Partner Interdependence Mediation Model
Source: Nurs Rep. 2025 Oct 20;15(10):372. doi: 10.3390/nursrep15100372 (PMC12567538; doi:10.3390/nursrep15100372)
Supplement: Supplementary file 1 [file nursrep-15-00372-s001.zip › Table S2.pdf]

Table S2. The Dyadic PCS and disability – depression – social support chain mediation effect (N=168).

Effect	Estimated value	SE	95%*CI*	*P* value
The actor effect of survivors				
Direct effect	-3.731	0.589	-5.014，-2.669	0.001
Indirect effect				
T0Disability⟶T1Survivor depression⟶T3SurvivorPCS	-0.066	0.165	-0.448，0.219	0.508
T0Disability ⟶T2Survivor social support ⟶ T3 Survivor PCS	0.019	0.059	-0.045，0.237	0.389
T0Disability ⟶T1 Survivor depression ⟶T2Survivor social support⟶T3Survivor PCS	-0.028	0.059	-0.182，0.065	0.487
T0Disability ⟶T1Spouse caregiver depression⟶ T3Survivor PCS	-0.210	0.194	-0.722，0.078	0.156
T0Disability ⟶T2Spouse caregiver social support⟶ T3Survivor PCS	0.062	0.079	-0.032，0.305	0.191
T0Disability ⟶T1Spouse caregiver depression⟶T2Spouse Caregiver social support⟶T3Survivor PCS	-0.085	0.077	-0.303，0.024	0.116
T0Disability ⟶T1 Survivor depression ⟶T2Spouse caregiver Social support⟶T3Survivor PCS	-0.026	0.027	-0.122，0.003	0.080
T0Disability ⟶T1Spouse caregiver depression⟶ T2Survivor social support⟶T3Survivor PCS	-0.019	0.038	-0.134，0.037	0.338
Total indirect effect	-0.353	0.237	-0.905，0.055	0.092
Total effect	-4.084	0.553	-5.219，-3.052	0.001
Partner effects of spouse caregivers				
Direct effect	-0.373	0.232	-0.836，0.073	0.107
Indirect effect				
T0Disability ⟶T1Spouse caregiver depression⟶T3Spouse caregiver PCS	-0.198	0.106	-0.468，-0.038	0.011
T0Disability ⟶T2Spouse caregiver social support⟶ T3Spouse caregiver PCS	-0.020	0.034	-0.141，0.021	0.279
T0Disability ⟶T1Spouse caregiver depression⟶ T2Survivor social support⟶T3Spouse caregiver PCS	0.028	0.039	-0.028，0.148	0.273
T0Disability ⟶T1 Survivor depression ⟶T3Spouse caregiver PCS	0.082	0.063	-0.011，0.245	0.080
T0Disability⟶T2Survivor social support⟶T3Spouse caregiver PCS	0.012	0.030	-0.022，0.131	0.354
T0Disability⟶T1Survivor depression⟶T2Survivor social support⟶T3Spouse caregiver PCS	-0.017	0.030	-0.104，0.029	0.342

T0Disability ⟶T1Survivor depression⟶T2Spouse caregiver social support⟶T3Spouse caregiver PCS	0.009	0.012	-0.005，0.049	0.181
T0Disability ⟶T1Spouse caregiver depression⟶ T2Survivor social support⟶T3Spouse caregiver PCS	-0.012	0.022	-0.081，0.015	0.321
Total indirect effect	-0.117	0.087	-0.332，0.018	0.093
Total effect	-0.490	0.231	-0.946，-0.057	0.030
