# Supplementary material for: The Interplay of Disability, Depression, Social Support, and Quality of Life in Middle-Aged and Young Couples Affected by Stroke: A Dyadic Path Analysis Using the Actor–Partner Interdependence Mediation Model
Source: Nurs Rep. 2025 Oct 20;15(10):372. doi: 10.3390/nursrep15100372 (PMC12567538; doi:10.3390/nursrep15100372)
Supplement: Supplementary file 1 [file nursrep-15-00372-s001.zip › Table S3.pdf]

Table S3. The Dyadic MCS and disability – depression – social support chain mediation effect (N=168).

**Effect**	**Estimated value**	**SE**	**95%*CI***	***P*-value**
The actor effect of survivors				
Direct effect	-1.598	0.530	-2.732，-0.641	0.001
Indirect effect				
T0Disability⟶T1Survivor depression⟶T3Survivor MCS	-0.502	0.287	-1.279，-0.096	0.007
T0Disability⟶T2Survivor social support⟶T3Survivor MCS	0.155	0.199	-0.183，0.639	0.302
T0Disability⟶T1Survivor depression⟶T2Survivor social support⟶T3Survivor MCS	-0.231	0.134	-0.643，-0.062	0.003
T0Disability⟶T1Spouse caregiver depression⟶T3Survivor MCS	0.087	0.190	-0.244，0.544	0.535
T0Disability⟶T2Spouse caregiver social support⟶T3 Survivor MCS	-0.064	0.084	-0.386，0.027	0.158
T0Disability⟶T1Spouse caregiver depression⟶T2Spouse caregiver social support⟶T3Survivor MCS	0.088	0.094	-0.015，0.399	0.100
T0Disability⟶T1Survivor depression⟶T2Spouse caregiver social support ⟶T3Survivor MCS	0.027	0.032	-0.003，0.142	0.089
T0Disability⟶T1Spouse caregiver depression⟶T2Survivor social support⟶T3Survivor MCS	-0.156	0.117	-0.515，-0.023	0.012
Total indirect effect	-0.597	0.355	-1.383，0.002	0.051
Total effect	-2.195	0.621	-3.458，-1.007	0.001
Partner effects of spouse caregivers				
Direct effect	-1.001	0.351	-1.727，-0.325	0.003
Indirect effect				
T0Disability⟶T1Spouse caregiver depression⟶T3 Spouse caregiver MCS	-0.212	0.170	-0.657，0.030	0.090
T0Disability⟶T2Spouse caregiver social support⟶T3 Spouse caregiver MCS	0.048	0.061	-0.021，0.260	0.167
T0Disability⟶T1Spouse caregiver depression⟶T2Spouse caregiver social support⟶T3 Spouse caregiver MCS	-0.066	0.066	-0.276，0.012	0.095
T0Disability⟶T1Survivor depression⟶T3 Spouse caregiver MCS	-0.058	0.138	-0.386，0.175	0.545
T0Disability⟶T2Survivor social support⟶T3 Spouse caregiver MCS	0.044	0.064	-0.051，0.228	0.288
T0Disability⟶T1Survivor depression⟶T2Survivor social support⟶T3 Spouse caregiver MCS	-0.066	0.050	-0.207，0.000	0.049
T0Disability⟶T1Survivor depression⟶T2Spouse caregiver	-0.020	0.020	-0.099，0.002	0.070

social support⟶T3 Spouse caregiver MCS				
T0Disability⟶T1Spouse caregiver depression ⟶ T2Survivor social support⟶T3 Spouse caregiver MCS	-0.045	0.036	-0.163，-0.002	0.038
Total indirect effect	-0.374	0.236	-0.963，0.004	0.052
Total effect	-1.375	0.412	-2.261，-0.650	0.001
